# A study of lymph node ratio in stage IV colorectal cancer

**DOI:** 10.1186/1477-7819-6-127

**Published:** 2008-12-01

**Authors:** Kristoffer Derwinger, Bengt Gustavsson

**Affiliations:** 1 Department of Surgery, Sahlgrenska University Hospital/Östra, Gothenburg, Sweden

## Abstract

**Background:**

The finding of metastasis in colorectal cancer, stage IV disease, has a major impact on prognosis and treatment strategy. Known important factors include the extent of the metastasis and the patients' performance status. The lymph node factors are of known importance in earlier cancer stages but less described in metastatic disease. The aim of the study was to evaluate lymph node status and ratio as prognostic markers in stage IV colorectal cancer.

**Methods:**

The study was retrospective and assessing all patients operated, with bowel resection, for an initial stage IV colorectal cancer during 1999–2003 (n = 136). Basic demographic data as well as given treatment was assessed. The Lymph node ratio (LNR), the quota between the number of lymph node metastasis and assessed lymph nodes, was calculated. LNR groups were created by ratio thirds, 3 equally sized groups. The analysis was made by LNR group and by eligibility for chemotherapy with cancer specific survival as outcome parameter.

**Results:**

The median survival (CSS) for the entire group was 431 days with great variability. For the patients eligible for chemotherapy it ranged from 791 days in LNR-group 1 to 433 days for the patients in group 3. For patients ineligible for chemotherapy the corresponding figures were 209 and 91 days. The eligibility for chemotherapy was a major prognostic factor which also takes co-morbidity, age and performance status into consideration. The LNR (p < 0.01) and the tumour differentiation grade were also significant (p < 0.05) factors regarding survival. The LNR group 3 was also associated with a higher frequency of multiple metastasis locations (p < 0.05) and of more side effects with chemotherapy and thus of reductions in dosage or pre-emptive treatment ending (p < 0.05).

**Conclusion:**

Stage IV colorectal cancer is a heterogeneous group regarding the survival prognosis. The lymph node ratio was found to be a significant marker for the survival prognosis (p < 0.0049). High and low risk groups could be identified with a survival difference of up to one year. It could be of importance when planning a treatment strategy or evaluating clinical data materials. A pathology report should include a node assessment even at presence of synchronous metastasis.

## Background

In Sweden about 5500 new colorectal cancers are diagnosed each year [1]. It is one of the more common forms of cancer and the incidence is slowly increasing. The main form of treatment is the surgical removal of the tumour. Preceding the operation there are preoperative investigations. The aim is ruling out findings that can alter the treatment strategy such as extra-intestinal manifestations or locally advanced tumours. The staging procedure is continued intra-operatively and ultimately completed post-operatively by the pathologists' analysis [2,3]. The cancers are staged and classified according to the UICC/AJCC standards of the TNM-system [4]. Almost 20% of the patients are of stage IV disease, characterized by either distant metastasis or by local overgrowth to adjacent organs, at the time of diagnosis [5].

An accurate cancer staging is not only a foundation in deciding treatment strategy but also an important prognostic tool [6]. When metastases or locally advanced growth are found there are several options that normally are discussed in a multidisciplinary team conference. These include the indication and timing of both surgery and chemotherapy and also the possible treatment of the metastasis. There is also the decision if it is possible to try for a curative intent or if a palliative strategy is the only option. There are indications for surgical resections, such as bleeding and obstruction, even in the palliative situation. All available prognostic information that can aid in this strategic decision-making is of clinical importance. Major surgery can have negative effects for the patient and the risk should be considered against the chance of potential benefit. The decisions are normally re-evaluated as the time and treatment progress and new data can get available.

There are known prognostic factors in stage IV disease such as the patients performance status, the number of metastatic organs and the tumour differentiation grade [5]. In the earlier colorectal cancer stages (I-III) there is a great prognostic interest in the lymph node assessment and status. Different lymph node related factors as size, distribution, numbers and even the number of assessed lymph nodes are considered as possible aids in prediction of prognosis [5,7,8]. Another possibility is by the lymph node ratio which is highly significant in stage III disease [9,10]. One problem is that these are only available after surgical resections. However, when available, they could add information and assist in the reassessments before the continued treatment. The lymph node factors are not as well studied in stage IV and also less frequently reported. The aim of the study was to evaluate lymph node status and ratio as prognostic markers in stage IV colorectal cancer.

## Methods

At the department of surgery, Sahlgrenska/Östra University Hospital, Gothenburg we are continuously making a registration of detailed clinical and pathological data. The registration is consecutive since 1999 for all patients treated at our unit for colorectal cancer. The study and registration was approved by the local Ethics committee and all the patients have given their written informed consent. Included in the database is also a continuous follow-up regarding treatment and survival. During the period 1999–2003, 198 patients were surgically treated for an initial stage IV colorectal cancer. The specimen had been assessed by the pathologist for the lymph node status in 136 patients, who were then included into the study. All had been operated with a surgical resection of the bowel tumour. We retrieved basic clinical parameters as gender, age, diagnosis, cancer location, performance status (PS) and type of operation. Treatment data with the use of chemotherapy, tolerability and side-effects was also considered as well as the number and location of the metastasis. We acquired the pathology data including the assessment of lymph nodes and differentiation grade. The lymph node ratio (LNR) was calculated as the quota between metastasis positive nodes and number of assessed lymph nodes. The LNR-groups were created by dividing the material into 3 equally sized groups, thus by ratio thirds, to have a possibility of identifying high/low risk groups. Survival data as well as treatment information was also retrieved. As the outcome parameter we used the cancer specific survival (CSS). Survival was assessed both by ratio groups and by eligibility for chemotherapy. A comparison was also made between LNR and N-status.

We used the JMP 4/SAS software for statistical analysis (SAS institute). The basic patient demographic data was set by distribution statistics with ANOVA or contingency tables for non-parametric statistics. The Kaplan-Meier method was used for univariate survival analysis and Log rank test was used to compare survival differences between the groups. The same analyses were made for TNM N-status as well as differentiation grade. We made a second analysis of the data for all patients who had had a full pathology assessment of at least 12 nodes as by UICC/AJCC recommendation to ensure validity. We also performed a Cox multivariate analysis including PS, differentiation grade, tumour location, age, given therapy, metastatic burden and also the lymph node factor. The later assessed both for the N-status and as LNR.

## Results

### The surgery and staging

The median age was 70 years with an equal gender distribution. The most frequently performed operations were the right hemicolectomy and the Hartman procedures. The most common indications for surgery in this patient group were bleeding or obstruction, the latter often resulting in resection and stoma formation. The preoperative work-up was done with chest x-ray and liver ultrasonography or CT-scan and were completed in 98% of elective cases. 7 patients had lung metastasis only and 87 patients had liver metastasis. An additional 14 patients had growth in both organs. Of the remaining 28 patients were 24 had emergency procedures and were classified as stage IV by an intra-operative finding of metastases. The remaining 4 patients were assessed as possible spread by the radiologist and confirmed as stage IV by the pathologist analysis from specimen and intra-operative biopsies.

### The pathology

The patient and pathology data are presented in table 1. The differentiation grade correlated significantly with the LNR group (p < 0.001). With a poor differentiation grade is was more common to have a higher number of metastatic nodes and also higher ratios. This also showed in the distribution of TNM N1 and N2. The median number of assessed nodes was 10 with a median of 4 metastasis positive nodes. There were only 2 patients without discovered lymph node metastasis and both had had very few assessed lymph nodes. We also found that with higher LNR-group it was increasingly more frequent with multiple metastasis locations (p < 0.05).

**Table 1:** Patient demography and pathology by lymph node ratio group

**LNR group**	**Ratio/Node quota**	**N**	**Differentiation grade (well/med./poor)**	**N-status (N1/N2)**	**Lymph nodes (median)**		**Chemotherapy eligibility (yes/no)**
						
					Assessed	Positive	
**1**	0–0.15	46	4/36/6	46/0	10 (1–19)	1 (0–3)	27/19
**2**	0.16–0.65	45	3/30/12	15/30	10 (4–21)	4 (1–11)	23/22
**3**	0.66–1	45	0/14/31	6/39	9 (2–32)	8 (2–26)	27/18

**Total**	0–1	136	7/80/49	67/69	10 (1–32)	4 (0–26)	77/59

### Chemotherapy

The treatment strategy was discussed in a multidisciplinary team conference including the possible use of chemotherapy. 77 of the patients were eligible for chemotherapy. The main reasons for ineligibility for chemotherapy were age, concomitant disease or poor performance status. All chemotherapy was given postoperatively. The common first line regime was 5-FU and Leucovorin which accounted for more than 85% of the given treatments. Second line chemotherapy, mainly using Campto and Oxaliplatin regimes, were given to 47 patients. The main reasons for termination of chemotherapy were toxicity or progression of the disease. Only 7 patients, of whom the majority had single metastasis, were later treated for the liver metastasis by radiofrequency ablation or surgical resection. Only one achieved long-term survival. We also noted that higher LNR-group and foremost group 3 had significantly more problems during the chemotherapy (p < 0.05). This showed by more adverse effects, lower tolerability and more frequently early treatment termination.

### The survival prognostics

**Figure 1:**
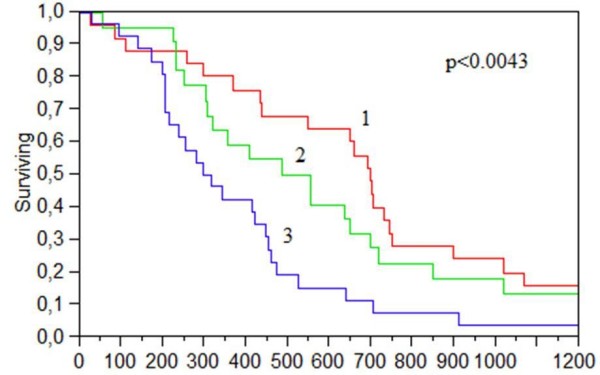
**Cancer-specific survival in stage IV colorectal cancer by lymph node ratio group 1–3**. Group 1 correspond to a low quota/ratio and 3 to a high ratio.

The median survival (CSS) for the entire group was 431 days with variability as shown in table 2. For the patients eligible for chemotherapy it ranged from 791 days in LNR-group 1 to 433 days for the patients in group 3. For patients ineligible for chemotherapy the corresponding figures were 209 and 91 days. In the univariate analysis the lymph node ratio was significant (p < 0.0049) were a higher quota corresponded with a worse prognosis (figure 1). The node status (N1–N2) had borderline significance (p < 0.06) for survival prognostics with N2 (more than 3 positive nodes) corresponding to a worse prognosis. The differentiation grade was also a significant factor (p < 0.001) where a poor grade corresponded to a worse prognosis. There were no significant differences in survival related to gender, diagnosis or cancer location. The eligibility for chemotherapy was highly significant (p < 0.001) but also contains factors as age and performance status. All survival results retained their significance when redoing the analysis for patients with at least 12 assessed nodes. In the Cox analysis the performance status and eligibility for chemotherapy was the most significant (HR 2.2 (1.1–4.3), p < 0.001) along with the differentiation grade (HR 2.0 (1.1–2.8), p < 0.05). Concerning the lymph nodes the LNR retained significance as a marker (HR 2.1 (1.3–3.6), p < 0.05) whilst the lymph node N-status was not significant.

**Table 2:** Survival by lymph node ratio group and therapy eligibility (median in days with upper/lower 95%)

**LNR group**	**Chemotherapy eligibility**		**All**
		
	Yes	No	
**1**	791 (538–864)	209 (144–757)	708 (298–824)
**2**	588 (349–745)	331 (271–514)	438 (346–688)
**3**	433 (281–488)	91 (26–173)	277 (173–473)

**Total**	538 (467–708)	229 (168–345)	431 (338–502)

## Discussion

The preoperative staging process has the aim of identifying the patients with metastatic disease. A positive finding has a major impact on the individual and does often lead to changes in the treatment strategy. The findings are usually discussed at a multi-modal treatment conference [11]. The first decision is the role and timing of the different treatment options. It is in many instances based on the radiological picture combined with the general health of the patient. The commonly used treatment in stage IV disease is chemotherapy, either as a palliative regime or as an attempt to downstage the tumour in preparation for surgery [12]. A strategy with curative intention can be attempted for some patients. There are chances of long-term survival and especially if the metastasis is solitary [13]. The liver metastasis can be treated by hepatic resections or radio-frequency ablations and pulmonary metastasis can be operated if unilateral [14].

A problem in the preoperative assessment is that a metastatic growth must be of a certain size to be detectable by radiology. Thus, there could be an uncertainty about the total tumour situation. The treatment decisions are often re-evaluated as new data gets available. The response to chemotherapy, with a possible down-staging effect, and the postoperative pathology report are among these important data. There are several known prognostic factors that should be considered [15]. The patients' general health and performance status are important along with the associated eligibility for chemotherapy. The CEA levels and the tumour differentiation grade can also be of interest [5,16,17]. The decision-making should balance the possibility of gaining long-term survival against the risk of complications, worsening the outcome and decrease in quality of life.

The most common role of surgery in stage IV is local control. The indications are then to prevent profuse blood-loss, to relieve obstruction or as a removal of mass [18,19]. A drawback is the associated risk of complications and the possibly prolonged hospital stay [20]. This has led to an increasing use of stents and thus a possibility of relieving obstruction without surgery [21]. For the patients that are ineligible for chemotherapy the prognosis is very poor (table 2). This concurs with our data and supports the idea that the non-surgical options are preferable for this group. Another important surgical indication is as the first step in an attempt to achieve long term survival. For colon cancer it is often preferable to start with the removal of the bowel tumour. The metastasis is then treated either simultaneously or as a second procedure. After surgery we gain access to more information about the tumour status through the pathology report.

Included in the pathology report are the tumour differentiation grade and also the lymph node assessment. The lymph node related factors are well described in the earlier cancer stages but less explored in stage IV. The lymph node ratio has been shown to a significant marker for the prognosis within stage III disease [10,22]. The ratio is a continuous variable but a grouping makes it more defined and facilitates the identification of risk groups. It could possibly give an indication on the tumour biology and thus also the total cancer burden. In our material we found a significant difference in median survival between LNR-groups 1 and 3 of almost one year. We noted that the possible long term survivors were mainly from LNR-group 1.

The lymph node ratio can be seen and used as a prognostic indicator. In our opinion a high quota can indicate a high risk that there is more of a disseminated disease. Thus, LNR-group 3 (with high quota) could be seen as a marker of having a higher risk of a metastatic growth that is not yet detectable by radiology. A high ratio also correlates to a higher risk of multiple metastasis locations and to a worse differentiation grade and often worse response to chemotherapy. All these factors add up towards a worse prognosis. This can then lead to a high risk of early "recurrence" and worse response to treatment. The prognostic indication could be an aid in the decision of the continued treatment. As discussed above it is possible to surgically treat the metastasis. However, it is not without risk and should be carefully. Thus in our opinion the possible candidates for curative intent should mainly be recruited among the patients with low lymph node quotas, since they could be more likely to benefit from the procedure.

As the LNR is a computation we would not call it a factor in itself. The ratio figures can dependent on the number of nodes assessed. The data and specific arithmetical numbers of a centre can thus often be unique. However, there will still be a distribution among them which can range from good to worse. The UICC/AJCC recommendation for the assessment is set at 12 nodes. In an effort to comply with this difficulty we did a second analysis, looking at the patients with at least 12 assessed nodes. That the result retained significance does strengthen the hypothesis that there is important prognostic information in the lymph node data. We believe that this material shows that there is information of importance in the node assessment even in stage IV and that the data should be requested. Interestingly, the N-status was not significant whilst the ratio was. An explanation could be that the latter also is affected by the differentiation grade. Another reason could be that the N2 only marks more than 3 positive nodes and thus lack the possibility to distinguish further details.

In our opinion, this material can show that the lymph node ratio could give an indication of the prognosis also in stage IV colorectal cancer. It is a rather simple method for getting a prognostic hint. However, in this heterogeneous group we do not want to point out or promote only one single factor. Rather we want to show how important the multidisciplinary approach is and that there is a large amount of information to be considered for treatment decisions. The inherent survival variability is great within the stage group. It is not fully covered within the limitations of the TNM-system and modifications have been suggested [23]. Additional pathology information, including lymph node data, would be of interest when reporting from treatment studies in this patient group. It could then provide further details about the patient selection and how we can interpret the new knowledge. One weakness in our material is the relatively small number of patients and that the study is retrospective. However, we believe this to be well compensated by the fact that the material is unselected and population based. All were included, registered and treated using the same guidelines.

## Conclusion

Stage IV colorectal cancer is a heterogeneous group regarding the survival prognosis. The lymph node ratio was found to be a significant marker for the survival prognosis (p < 0.0049). High and low risk groups could be identified with a survival difference of up to one year. It could be of importance when planning a treatment strategy or evaluating clinical data materials. A pathology report should include a node assessment even at presence of synchronous metastasis.

## Competing interests

The authors declare that they have no competing interests.

## Authors' contributions

KD was involved in the concept and design, data collection, analysis and interpretation and preparation of the manuscript. BG was involved in the concept and design, data collection, analysis and interpretation and preparation of the manuscript. Both authors read and approved the final manuscript
